# How Biomarker-Based Diagnosis and Treatment Affect Alzheimer’s Stigma: Results of a Randomized Trial

**DOI:** 10.1093/geroni/igab046.926

**Published:** 2021-12-17

**Authors:** Shana Stites, Jeanine Gill, Emily Largent, Kristin Harkins, Abba Krieger, Jason Karlawish

**Affiliations:** 1 University of Pennsylvania, Philadelphia, Pennsylvania, United States; 2 Wharton School of Business, Philadelphia, Pennsylvania, United States

## Abstract

Alzheimer’s disease (AD) causes progressive disability and, ultimately, death. Currently no therapy can delay or slow cognitive and functional decline. This prognosis contributes to the general public’s negative reactions—discrimination, pity, and social distance—toward individuals with AD and their families. But what if, using AD biomarker tests, diagnosis was made earlier and treatment was available? Stigma of AD might change. This project aimed to discover how diagnosis and treatment of AD before the onset of cognitive impairment would change public stigma, and how these effects might differ in ethnoracial populations. Comparisons of 12 experimental conditions (i.e., 2 (biomarker test result) x 2 (treatment availability) x 3 (cognitive impairment: none, mild, moderate)) are conducted in two independent samples of self-identified White (N=800) and Black (N=800) Americans. Findings anticipate the translation of the preclinical AD construct into care and will inform public policies and interventions to mitigate public stigma of AD.

